# Strategies to increase downloads of COVID–19 exposure notification apps: A discrete choice experiment

**DOI:** 10.1371/journal.pone.0258945

**Published:** 2021-11-01

**Authors:** Jemima A. Frimpong, Stéphane Helleringer

**Affiliations:** 1 Division of Social Science, Program in Social Research and Public Policy, New York University–Abu Dhabi (UAE), Abu Dhabi, United Arab Emirates; 2 Carey Business School, Johns Hopkins University, Baltimore, MD, United States of America; Mayo Clinic Minnesota, UNITED STATES

## Abstract

Exposure notification apps have been developed to assist in notifying individuals of recent exposures to SARS-CoV-2. However, in several countries, such apps have had limited uptake. We assessed whether strategies to increase downloads of exposure notification apps should emphasize improving the accuracy of the apps in recording contacts and exposures, strengthening privacy protections and/or offering financial incentives to potential users. In a discrete choice experiment with potential app users in the US, financial incentives were more than twice as important in decision-making about app downloads, than privacy protections, and app accuracy. The probability that a potential user would download an exposure notification app increased by 40% when offered a $100 reward to download (relative to a reference scenario in which the app is free). Financial incentives might help exposure notification apps reach uptake levels that improve the effectiveness of contact tracing programs and ultimately enhance efforts to control SARS-CoV-2. Rapid, pragmatic trials of financial incentives for app downloads in real-life settings are warranted.

## Introduction

Contact tracing is an important intervention to control the COVID–19 pandemic. It entails interviewing newly diagnosed persons to obtain lists of the individuals they have recently interacted with. A trained health worker then attempts to notify these “contacts” of their possible exposure, for example by phone or during a home visit. This gives an opportunity to encourage at-risk contacts to isolate and undergo testing. This traditional approach to contact tracing is however time and labor-intensive [1]. It might reach a limited number of contacts. It might also reach them after they have further transmitted SARS-CoV-2 [2].

Newly developed digital tools might remedy some of these limitations of traditional contact tracing programs [3]. Exposure notification apps (“EN apps”, thereafter) might alert additional contacts faster about their potential exposure. EN apps use Bluetooth technology to keep logs of contacts between app users in real time [4]. Once app users are diagnosed with (or show symptoms suggestive of) COVID–19, they can voluntarily enter this information on the app. Other users recorded in their contact logs are then instantaneously notified and encouraged to self-isolate. Several technology firms have developed infrastructure to facilitate the deployment of EN apps, and different versions of EN apps are being used around the world. Several evaluations suggest that the roll-out of EN apps might have prevented infections and deaths in various settings [5, 6].

The impact of EN apps on contact tracing outcomes depends on adoption of the app among potential users. Even though EN apps might help reduce the spread of SARS-CoV-2 at low levels of app uptake [7], a critical mass of mobile subscribers is required to effectively interrupt transmission chains. According to some models, as many as 80% of mobile subscribers were required to use the app, in order to achieve epidemic control prior to the roll-out of vaccines [2]. However, such levels of uptake have not been reached [8].

Most approaches to improving app uptake focus on providing information about how EN apps function [9], addressing concerns about data privacy [10] and/or improving the accuracy of exposure notifications generated by an app [11–14]. In opinion surveys, the intentions of potential app users to download an EN app were particularly influenced by the app’s true positive rate (i.e., its “sensitivity”) in detecting exposures to SARS-CoV-2 [11]. Other aspects of EN apps that might influence decisions to download include technical issues such as concerns about data consumption, the availability of storage space on users’ phone [15], as well as trust in government and institutions implementing the EN app [16–18].

The adoption of health-related innovations such as EN apps can also be accelerated by offering financial incentives to potential users [19]. Incentives are payments that are conditional on specific actions or behaviors. Patients and at-risk populations are often responsive to financial rewards that encourage adopting healthy behaviors [20] or undergoing specific diagnostics and procedures [21]. For example, financial incentives helped increase engagement in smoking cessation programs in multiple settings and population groups [22–24]. They have been effective in increasing the uptake of HIV testing and other HIV prevention tools in at-risk populations [25–29]. They have also helped promote participation in data collection [30, 31].

The potential effects of financial incentives on the download rates of EN apps have however seldom been investigated, since the beginning of the COVID-19 pandemic. In a recent study in Germany, financial incentives of up to 5 euros (approximately 5.60 US dollars) increased app downloads among an online panel of participants [9]. However, such small incentives might lead to “peanuts effects” [32] in other settings, where they might be perceived as trivial by potential users.

In May 2020, we conducted a discrete choice experiment to assess multiple approaches to increasing the uptake of EN apps: improving app accuracy, strengthening privacy protections and/or offering financial incentives to potential users. A discrete choice experiment is a survey methodology in which respondents choose between hypothetical versions of a good or service, characterized by a small number of randomly selected attributes [33]. In this DCE, we explored a broad range of potential financial incentives to download an EN app, including one-time payments of up to 100 US dollars.

## Data and methods

### Data source

The study was approved by the institutional review board of the Johns Hopkins Bloomberg School of Public Health. It was conducted on May 28–29, 2020. We recruited a sample of internet users who resided in the United States, via the Qualtrics online panel. Qualtrics is a commercial firm that specializes in providing services to businesses and organizations, including tools for survey research. We entered a service provider agreement with Qualtrics for the conduct of this discrete choice experiment (based on a set price per completed survey). Specifically, we used the Qualtrics online panel to recruit participants. This is a frequently used option for the constitution of convenience online samples, including in social science and health research [34]. This online panel is constituted of potential respondents, who have signed up to take online surveys in exchange for various incentives and gifts. We indicated our target sample size and eligibility criteria to Qualtrics, then the firm contacted select participants in the online panel to alert them to our study. Qualtrics also handled the compensation of participants who completed the discrete choice experiment. We do not know the exact value of the incentive/gift study participants received from Qualtrics. However, the total cost of the survey per interview amounted to 7$.

To be eligible, potential participants had to a) be aged 18–69 years old, b) own a smart phone, c) read and understand English, and d) report never having tested positive for SARS-CoV-2. We excluded people aged 70 years and older because mathematical models of the potential effects of an EN app in the early phases of the COVID-19 pandemic [2] have assumed that individuals in that age group will rely on other preventive measures (e.g., social distancing) to limit exposure to SARS-CoV-2. We excluded individuals with positive test results because few cases of reinfection among recovered COVID-19 patients had been documented by May 2020 [35]. We thus assumed that most internet users with positive test results would have developed (temporary) immunity to the virus that was circulating at the time of the study. Since EN apps aim to detect contacts during which SARS-CoV-2 can be transmitted, we assumed that this “recovered” group would not benefit from notifications generated by the app. Informed consent for study participation was obtained online: potential participants read the text of the consent and clicked a checkbox noting that they understood the consent form and agreed to participate in the study.

### Experimental design

we asked respondents to make 10 choices between two hypothetical EN apps, each defined by 6 attributes (Table 1). We selected these attributes and their levels based on a review of the literature on contact tracing for COVID–19 [1], expert consultations, and descriptions of Bluetooth-based EN apps [4, 36].

**Table 1:** Strategies, attributes and levels included in the discrete choice experiment.

Strategy to improve uptake of EN app	Attributes	Levels
Improving Accuracy	False notifications	1 in 100 notifications received from the app is an error
		5 in 100 notifications received from the app are errors
		15 in 100 notifications received from the app are errors
	Sensitivity	You are notified about 60% of your contacts with infected app users
		You are notified about 80% of your contacts with infected app users
		You are notified about 95% of your contacts with infected app users
Strengthening Privacy	User details	App does not ask for user details (health dept. cannot contact you)
		App asks for phone number or email (health dept. can contact you)
	Location	App does not collect any location data
		App asks user for zip code
		App tracks location (by GPS)
	Data sharing	You make your own COVID status available only to other app users
		You make your own COVID status available to health department
		You make your own COVID status and list of contacts available to health department
Offering financial incentives	Price/incentive to download	User pays $4.99
		App is free
		User gets $10
		User gets $50
		User gets $100

Notes: COVID status refers to the COVID–19 status of the user, as determined by test results and/or reported symptoms.

Three of these attributes were related to privacy features: (1) whether the EN app asks users to provide their phone number or email at download, (2) whether the EN app collects location data (e.g., through GPS tracking), and (3) whether users share their anonymized data with other app users or the health department. Two of the attributes concerned the accuracy of the EN app: (4) the rate at which the app makes errors in notifying users of SARS-CoV-2 exposure (“false positive rate”), and (5) the proportion of exposures to other app users who carry SARS-CoV-2 that are notified to users (“true positive rate” or sensitivity). The final attribute was (6) the price or incentive that a user might pay or receive for downloading the EN app.

We selected the different EN apps presented to respondents at random among 810 possible app configurations resulting from combinations of app attributes (Table 1). We used a fractional factorial design to generate the packages of 10 choice sets shown to each respondent [37]. Specifically, we used a balanced overlap design, which presents respondents with different packages of choice sets [37]. In total, we created 260 versions of choice packages, which were then randomly allocated to respondents. This approach ensures that the different levels in Table 1 are extensively represented in the experiment. It does not place a disproportionate weight on some potential levels or attributes of an EN app [37].

In each choice set, the two EN apps were labeled as “app 1” and “app 2”. Respondents also had the option not to download any of the proposed apps. This ‘opt-out’ option increases the external validity of discrete choice data [38]. Based on standard sample size calculations, we required 250 respondents to detect the main effects of attribute levels in Table 1 [39].

Before starting the experiment, respondents were provided with explanations about contact tracing and EN apps. In doing so, we set explicit parameters for characteristics of an EN app that were not represented in our experimental design: for example, we instructed respondents to assume that using EN apps would not count towards their monthly data usage.

We pre-tested the online survey with a few collaborators (n = 6). This allowed addressing technical issues in our survey form, as well as eliciting initial feedback on the wording of questions and instructions. Based on feedback obtained during this pre-test, we produced a revised version of our online survey. Then, we conducted an online pilot of this revised version with 50 respondents recruited through the Qualtrics online panel. In that pilot, we encouraged participants to use open-ended fields to express their feedback on study questions and instructions. Both investigators reviewed the pilot data. We then revised the online survey to address reported issues. Respondents who completed the online pilot are not included in our analyses.

### Data quality

we included an attention check [40] prior to the discrete choice experiment. Respondents who failed that attention check were excluded from completing the survey. We evaluated respondents’ understanding of the explanations about contact tracing and EN apps using six “quiz” questions, after which we provided them with feedback about the right answers.

### Statistical analyses

We used data on age reported during screening to describe the selectivity of the consent process and attention checks among eligible internet users. Unfortunately, other characteristics (e.g., gender, race/ethnicity) were not assessed at the screening stage. We thus could not assess other dimensions of sample selectivity. We then described the demographic characteristics of participants who completed the discrete choice experiment, as well as their perceptions of the health threat posed by COVID–19 at the time of the study. We analyzed the discrete choice data using random parameter logit models, which allow preferences for app attributes to vary across respondents [41]. Using estimates from these models, we measured the relative importance of app attributes in explaining decisions to download an EN app [42]. We then calculated predicted probabilities to assess the potential impact of financial incentives on download rates [43]. All analyses were conducted in STATA 15.1 using the mixlogit, mixlbeta and mixlpred commands [44].

## Results

**Fig 1:**
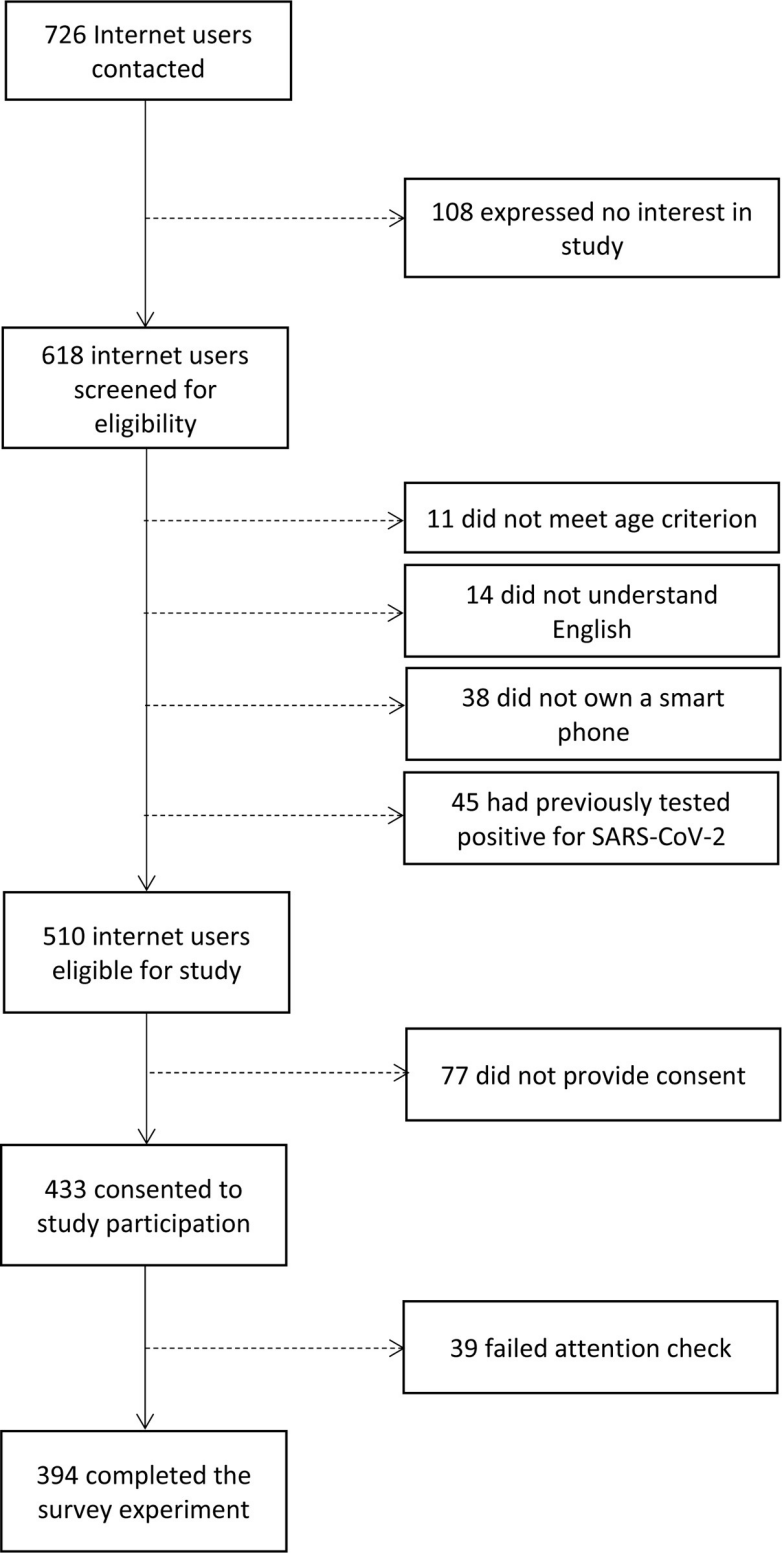
Flow chart of the study enrollment process.

We contacted 726 internet users (Fig 1). One hundred and eight internet users immediately stated that they were not interested in learning more about the study and were not screened for eligibility. Among the 618 internet users who were screened for eligibility, 11 did not meet age requirements, 14 reported not understanding English, 38 reported that they did not own a smart phone and 45 reported recently testing positive for SARS-CoV-2. They were thus excluded from the study. In total, 510 internet users were eligible for study participation. Seventy-seven potential participants refused to provide consent (77/510, 15.1%). An additional 39 participants failed the attention check inserted in the survey and were excluded from the study at that time (39/510, 7.6%). A total of 394 participants met the eligibility criteria, consented, and passed the attention check.

**Fig 2:**
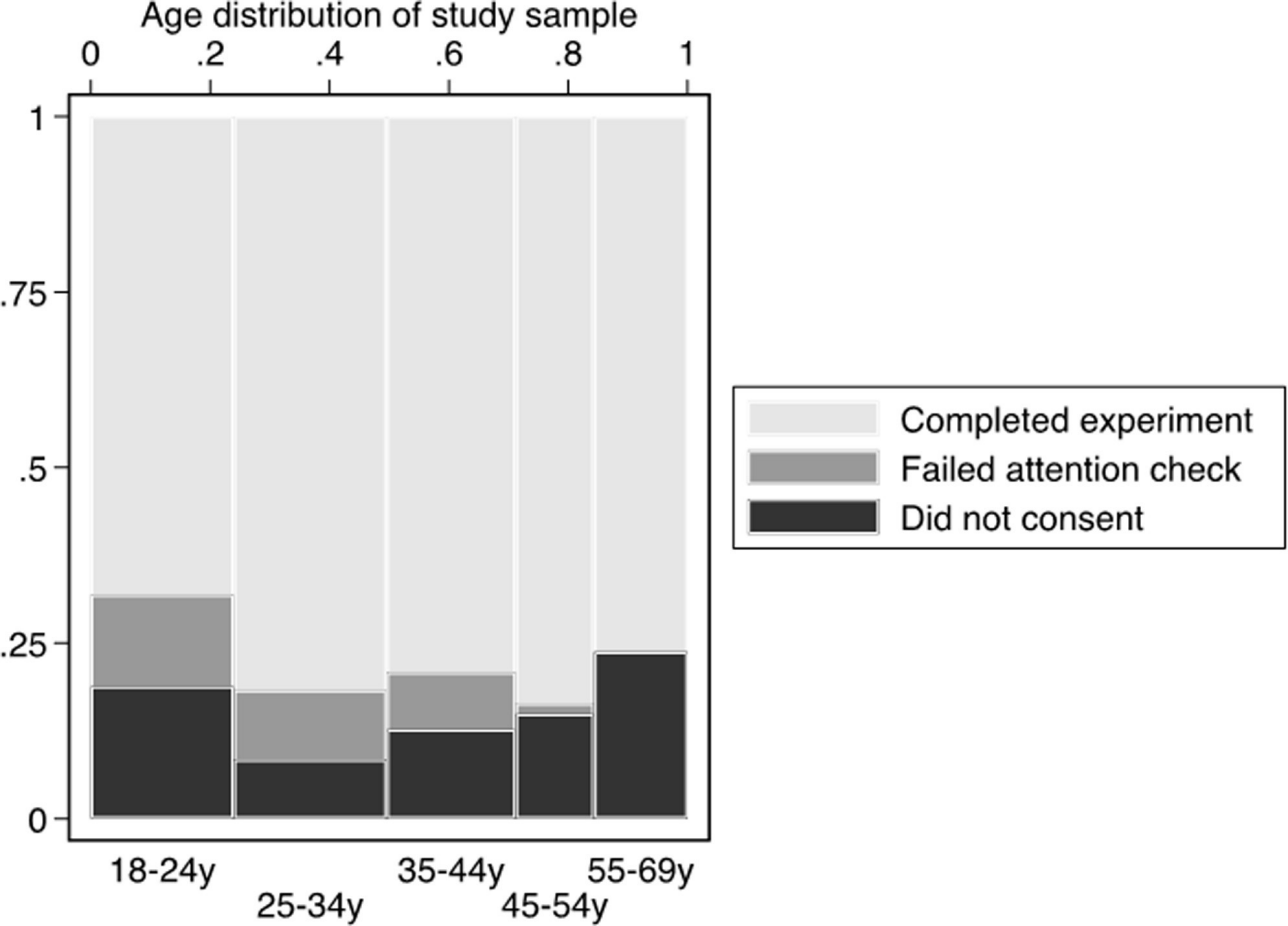
Age-related selectivity of study sample (n = 510). Notes: The width of each bar represents the proportion of the study sample included in each age group.

Among eligible internet users, participation outcomes varied by age (Fig 2). Non-consent was highest among 18–24 years old (23/122, 18.9%) and 55–69 years old (19/80, 23.8%). It was lowest among 25–34 years old (11/131, 8.4%). Younger respondents were also more likely to be excluded from the study because they failed the attention check. For example, 16 of 122 eligible 18–24 years old failed the attention check (13.1%), whereas only one of the 67 eligible 45–54 years old (1.5%) and none of the 55–69 years old failed this check.

The demographic characteristics and risk perceptions of the 394 participants are presented in Table 2. The sample was predominantly women (n = 268, 68.0%). One in 5 respondents belonged to the 18–24 years old age group, whereas only 3.8% were 65–69 years old. Approximately half of the respondents were non-Hispanic whites (210/394, 53.3%), but other groups were also represented including non-Hispanic blacks (54/394, 10.8%) and Hispanics (90/394, 22.8%). One in 3 respondents had completed college or higher, whereas 1 in 4 had a high school degree or had not completed high school. Only 22 out of 394 respondents reported being “not at all worried” about COVID–19 (5.6%).

**Table 2:** Descriptive statistics.

Characteristics	N(%)
Gender	
Men	126 (32.0)
Women	268 (68.0)
Racial/ethnic group	
Non-Hispanic white	210 (53.3)
Non-Hispanic black	54 (13.7)
Hispanic	88(22.3)
Other racial/ethnic group	42 (10.7)
Age group	
18–24y	83 (21.1)
25–34y	107 (27.2)
35–44y	87 (22.1)
45–54y	56 (14.2)
55-64y	46 (11.7)
65-69y	15 (3.8)
Educational level	
High school or less	97 (24.6)
Vocational degree	13 (3.3)
Associates’ degree/incomplete college	139 (35.3)
Undergraduate degree	98 (24.8)
Post-graduate degree	47 (11.9)
COVID-related risk perceptions	
Extremely worried	152 (38.6)
Moderately worried	99 (25.1)
Somewhat worried	72 (18.3)
Slightly worried	47 (11.9)
Not all worried	22 (5.6)
Don’t know	2 (0.5)

Notes: Numbers in parentheses are column percentages.

Three respondents (0.8%) did not answer any of the quiz questions about EN apps correctly, whereas 81 respondents (20.6%) answered all questions correctly (S1 Fig). Out of 3,940 choices between hypothetical EN apps that were made during the experiment, study respondents selected the opt-out “no download” option 850 times (21.6%). Approximately half of respondents never selected the opt-out option (S2 Fig), whereas close to 10% selected this option at every choice set.

**Fig 3:**
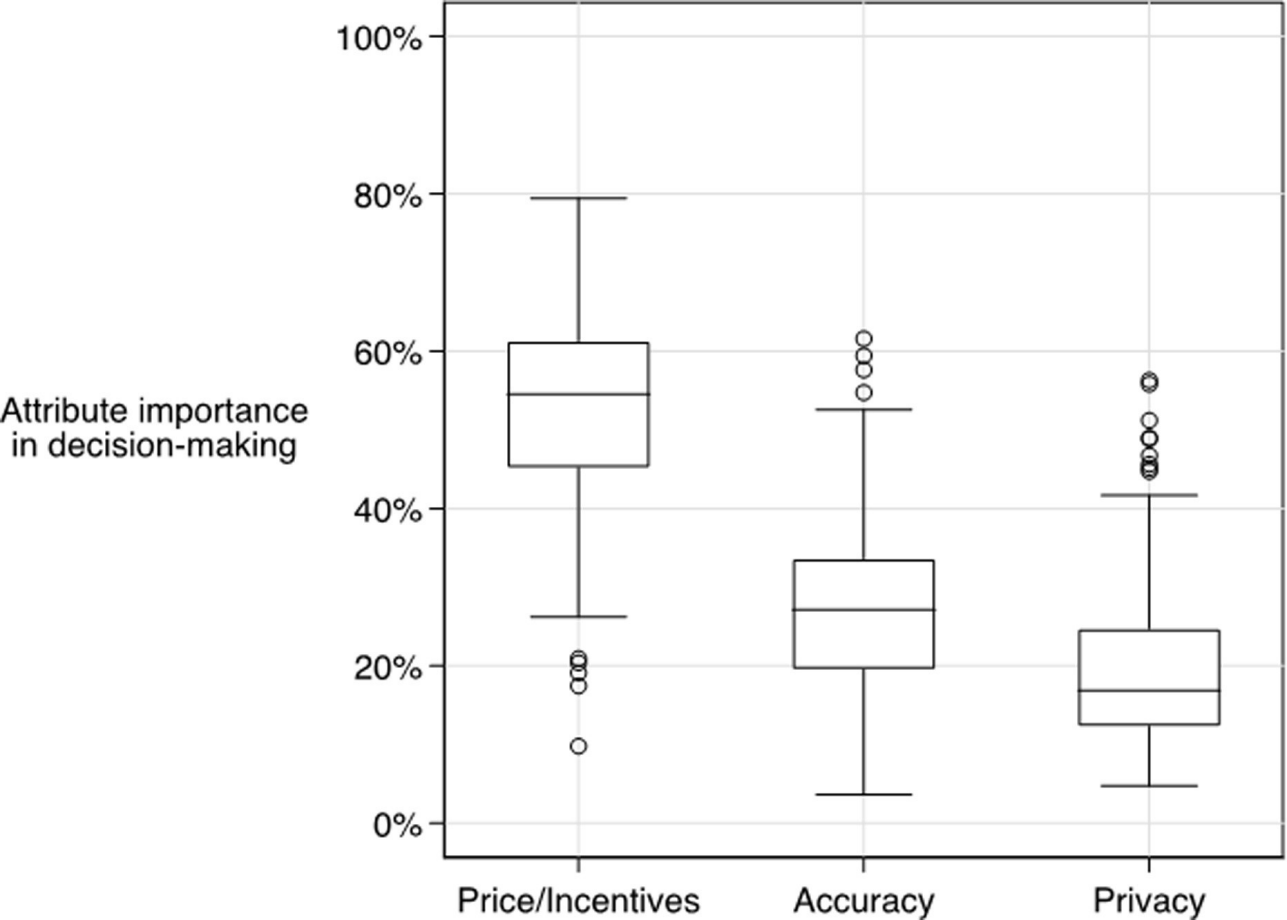
Relative importance of attributes in explaining decision-making about app downloads (n = 394). Notes: Attributes are grouped by strategy as in Table 1. The box plots represent the distributions of relative importance scores, across all individuals who completed the discrete choice experiment.

Based on results from random parameter logit models (S3 Fig), prices/incentives were twice as important as app accuracy and privacy protections in respondents’ decision-making about app downloads (Fig 3). Variations in price/incentive accounted for more than 50% of respondents’ decision-making, whereas attributes related to accuracy of the EN app in detecting exposures to SARS-CoV-2 accounted for approximately 25% of the decision-making to download an app. Attributes related to privacy features of the application accounted for less than 20% of respondents’ decisions.

**Fig 4:**
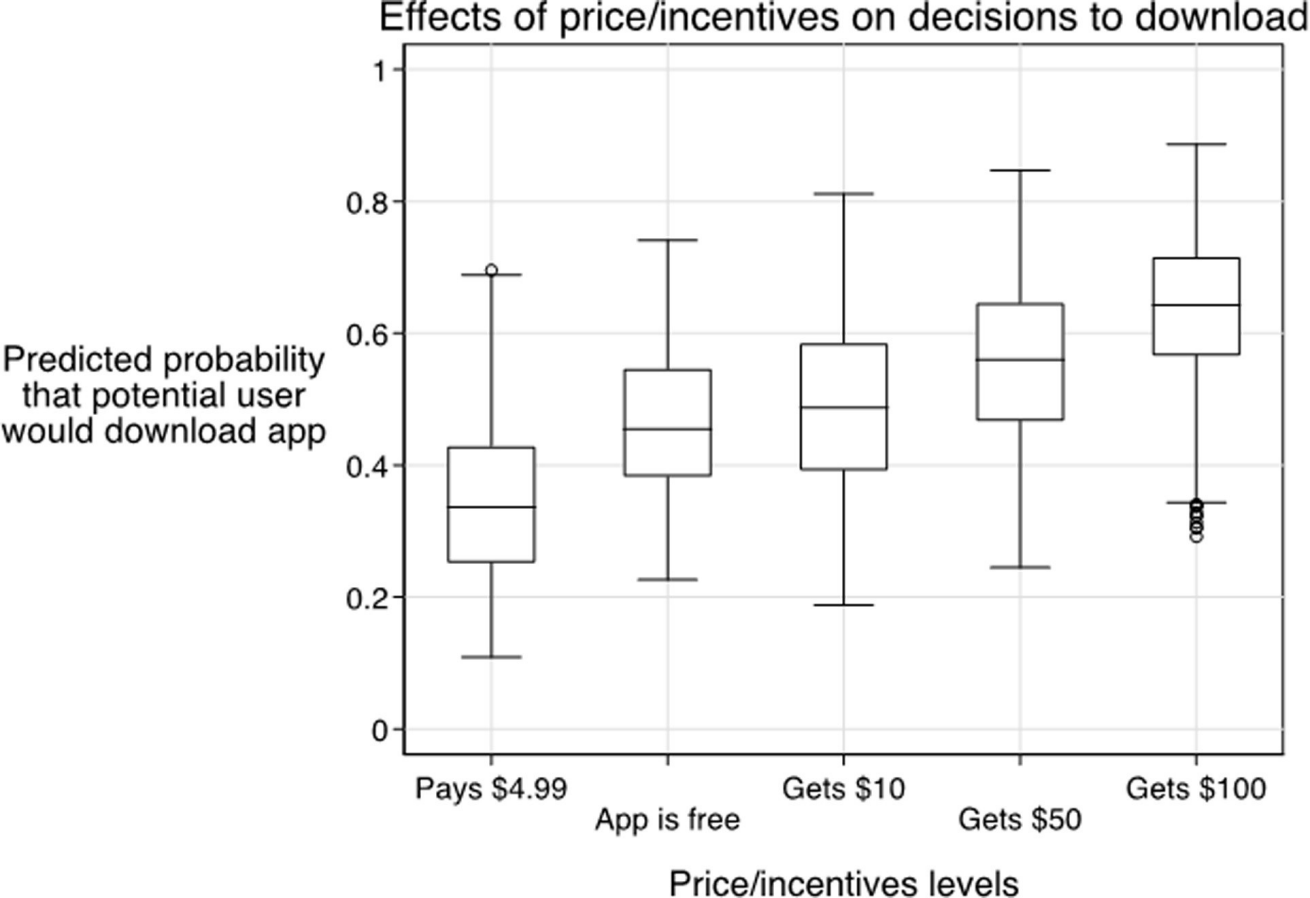
Predicted effects of price/incentives on decisions to download EN apps (n = 394). Notes: We used preference estimates from random parameter logit models (S3 Fig) to predict the probability that a participant would select a given app at different levels of price/incentives. The box plots represent the distributions of these predicted probabilities across respondents.

The predicted probability that potential users would download an EN app increased from 0.34 when the app cost $4.99 to download to 0.46 when free. The probability of downloads increased to 0.56 and 0.64 when potential users were given $50 or $100 incentives, respectively (Fig 4).

## Discussion

We compared the relative importance of three key strategies to accelerate uptake of exposure notifications apps for COVID–19. Among these strategies, financial incentives had the largest potential effects. In the context of this survey-based discrete choice experiment, offering a $100 incentive to download an EN app resulted in a 40% increase in the probability to download, compared to a situation in which the app was free (from 0.46 to 0.64). Other attributes of EN apps such as their privacy protections and accuracy in detecting exposure to SARS-CoV-2 also influenced decisions of potential users, but to a lesser extent.

Our study has several limitations. Our estimates of the importance of price/incentives in decisions to download EN apps pertain to the context of our experiment. In another experimental set-up in which there are no levels requiring potential users to pay for the EN app, the importance of prices/incentives in the decision-making process may be attenuated. We conducted the discrete choice experiment among a convenience online sample, which is not representative of the population of potential EN app users in the US. Furthermore, participation in the experiment was rewarded by an incentive or gift provided by Qualtrics. As a result, our convenience sample might have included participants who are more responsive to financial incentives than other internet users or population members. In addition, some of the respondents might have found explanations about contact tracing and EN apps provided prior to the experiment, difficult or very difficult to understand. However, in real-world settings, significant numbers of potential users might make decisions about downloading EN apps based on incomplete and potentially misunderstood information. In robustness tests however, we repeated study analyses after excluding these groups. We obtained similar estimates of attribute importance.

We provided information about certain levels and characteristics of EN apps in ways that might have been difficult for respondents to grasp. For example, we expressed sensitivity of the EN app as percentages, rather than counts (as we used for false negatives, see Table 1). Our experiment did not present respondents with app configurations in which they did not have to share their personal data to receive exposure notifications. Such options might be appealing to potential users with strong privacy concerns. They might however lead to free-riding behaviors, and limit the overall effectiveness of EN apps and its expected societal benefits [45, 46].

Importantly, we did not investigate interactions between attributes. For example, respondents may express stronger preferences for financial incentives when the proposed EN app is less sensitive or yields additional false notifications. Some population groups may also be more responsive to financial incentives than others. We did not investigate such interactions due to our limited sample size, and the selectivity of our study sample.

Data from discrete choice experiments might also be affected by hypothetical bias: what people indicate they would do during an experiment might differ from the choices they will make in real-life conditions [47]. In an online experiment in Germany, for example, financial incentives generated an increase in the uptake of EN apps that was smaller than the increase in willingness to download such apps reported by participants in hypothetical survey questions [9].

Finally, our study focused solely on the one-time decision to download an EN app, even though the effects of EN apps on epidemic dynamics depend on longer-term commitments to use these apps and their functionalities [2, 6]. Financial incentives and other rewards might need to be offered repeatedly to EN app users to sustain use of an EN app over time and/or to encourage repeated interactions with the app (e.g., reporting symptoms or test results). In other areas of health (e.g., HIV treatment, behaviors related to non-communicable diseases), however, financial incentives have had mixed effects in sustaining healthy behaviors over long periods of time [48–50].

Additional research is also needed, as the optimal ways to deliver financial incentives for EN apps remain unclear. This includes, for example, refining the amount of payments to offer to potential users. Incentive schemes should avoid “peanuts effects” [32], which emerge when amounts are set too low to motivate potential users. Given the expected societal benefits of COVID–19 control (e.g., economic recovery), high financial incentives (e.g., $100 or more) might remain cost-effective and should be considered. However, incentive amounts should not be set so high that some potential users with financial needs cannot effectively decide not to download the app [51]. If designed within a health equity framework [3], incentive schemes could also help alleviate (some of) the major disparities in the burden of COVID–19 documented in the US and elsewhere [52]. The modalities through which incentives are paid to potential users (e.g., gift cards, tax credits) might also modify the effects of such incentives on the uptake of EN apps. In an experiment in Italy, potential blood donors were reluctant to accept cash payments for a donation, but frequently accepted vouchers of the same amount [53].

There is also a risk that providing financial incentives to download EN apps might “crowd out” more altruistic reasons for using EN apps, attract malicious users and ultimately limit the adoption of EN apps and their effectiveness. Such negative effects of financial incentives have been observed in several prosocial behaviors [54], i.e., behaviors that primarily benefit others or society as a whole, rather than the person adopting the behavior, who might even incur a cost [55]. For example, in the context of blood donations, financial incentives did not seem to crowd out more altruistic motives for giving blood. They have thus been recommended as an important to avoid shortages in the blood supply [56]. In the context of charitable donations, on the other hand, thank-you gifts provided to donors reduced donation rates, probably because they made more altruistic motives to donate less salient in the decision-making process of potential donors [54]. The potential for such unintended negative effects to affect the provision of financial incentives for downloading EN apps should be investigated.

Finally, our discrete choice experiment, and other trials of financial incentives for downloading EN apps [9], were conducted prior to the large-scale roll-out of vaccines against SARS-CoV-2/COVID-19, and the emergence of more transmissible forms of the virus. Many high-income countries have now also launched digital tools to monitor vaccination uptake and/or have adopted vaccination requirements to access various settings and places. Such new tools and risks might have modified attitudes and preferences towards EN apps. Future investigations of incentives to use EN apps for COVID-19 control should thus be carried out in populations with diverse vaccination status.

## Conclusion

The effectiveness of EN apps depends on reaching a critical mass of adopters in a reasonable time frame. It is unlikely however that heightened privacy protections and improved accuracy of EN apps will be sufficient to achieve levels of app uptake required to affect epidemic dynamics. Our work indicates that financial incentives to download might have large effects on the rate at which EN apps are adopted in populations affected by COVID–19. Rapid, pragmatic trials investigating the complex effects of financial incentives in real-life settings are now needed. If effective, such incentives might help EN apps reach uptake levels that improve the effectiveness of contact tracing programs and ultimately help increase the likelihood of controlling SARS-CoV-2.

## Supporting information

S1 Fig Distribution of answers to quiz questions to measure understanding of EN apps and instructions (n = 394). Notes: respondents were asked 6 questions to elicit their understanding. These questions were asked before the beginning of the discrete choice experiment.
(DOCX)

S2 Fig Selection of the opt-out “No download” option during the discrete choice experiment (n = 394). (DOCX)

S3 Fig Distributions of individual preferences for app attributes (n = 394). Notes: The plots represent distributions of individual estimates of preferences for attributes obtained from a random parameter logit model. They were computed using the mixlogit and mixlbeta commands in Stata, with 500 Halton draws. Estimates above the red dotted line indicate positive effects of attributes on respondents’ utility levels. Acronyms and abbreviations: DOH = Department of Health; Status refers to the COVID–19 status of the user, as determined by test results and/or reported symptoms.
(DOCX)

S1 File (ZIP)
