# Supplementary material for: Strategies to increase downloads of COVID–19 exposure notification apps: A discrete choice experiment
Source: PLoS One. 2021 Nov 1;16(11):e0258945. doi: 10.1371/journal.pone.0258945 (PMC8559927; doi:10.1371/journal.pone.0258945)
Supplement: S1 Fig — Notes: respondents were asked 6 questions to elicit their understanding. These questions were asked before the beginning of the discrete choice experiment. (DOCX) [file pone.0258945.s001.docx]

**S1 Fig:**

**Distribution of answers to quiz questions to measure understanding of EN apps and instructions (n=394).**

Notes: respondents were asked 6 questions to elicit their understanding. These questions were asked before the beginning of the discrete choice experiment.
