# Supplementary material for: Strategies to increase downloads of COVID–19 exposure notification apps: A discrete choice experiment
Source: PLoS One. 2021 Nov 1;16(11):e0258945. doi: 10.1371/journal.pone.0258945 (PMC8559927; doi:10.1371/journal.pone.0258945)
Supplement: S2 Fig — (DOCX) [file pone.0258945.s002.docx]

**S2 Fig:**

**Selection of the opt-out “No download” option during the discrete choice experiment (n=394).**
