# Supplementary material for: Strategies to increase downloads of COVID–19 exposure notification apps: A discrete choice experiment
Source: PLoS One. 2021 Nov 1;16(11):e0258945. doi: 10.1371/journal.pone.0258945 (PMC8559927; doi:10.1371/journal.pone.0258945)
Supplement: S3 Fig — Notes: The plots represent distributions of individual estimates of preferences for attributes obtained from a random parameter logit model. They were computed using the mixlogit and mixlbeta commands in Stata, with 500 Halton draws. Estimates above the red dotted line indicate positive effects of attributes on respondents’ utility levels. Acronyms and abbreviations: DOH = Department of Health; Status refers to the COVID–19 status of the user, as determined by test results and/or reported symptoms. (DOCX) [file pone.0258945.s003.docx]

**S3 Fig:**

**Distributions of individual preferences for app attributes (n=394)**

Notes: The plots represent distributions of individual estimates of preferences for attributes obtained from a random parameter logit model. They were computed using the mixlogit and mixlbeta commands in Stata, with 500 Halton draws. Estimates above the red dotted line indicate positive effects of attributes on respondents’ utility levels.

Acronyms and abbreviations: DOH = Department of Health; Status refers to the COVID–19 status of the user, as determined by test results and/or reported symptoms.
